# Traumatic transnasal penetrating injury with cerebral spinal fluid leak

**DOI:** 10.17179/excli2018-1971

**Published:** 2019-04-08

**Authors:** Tan Sui Teng, Noor Liza Ishak, Sethu Thakachy Subha, Saraiza Abu Bakar

**Affiliations:** 1 Department of Otorhinolaryngology, Head and Neck Surgery, Serdang Hospital, Jalan Puchong, Kajang, Selangor Darul Ehsan, 43000 Malaysia; 2 Ear, Nose and Throat Unit, Department of Surgery, Faculty of Medicine and Health Sciences, Universiti Putra Malaysia

**Keywords:** pencil, penetrating trauma, skull base fracture, CSF leak

## Abstract

CSF leak in penetrating skull base injury is relatively rare compared to close head injury involving skull base fracture. We report a 5-year-old boy presented with epistaxis and impacted pencil into the left nostril. The child was hemodynamically stable without any neurological deficit. Intraoperatively, there was a nasal septal defect posteriorly with anterior skull base fracture associated with CSF leak. The pencil was removed from the left nostril and the CSF leak was repaired using harvested abdominal fat under the same setting. Computed Tomography (CT) of the brain showed right cribriform plate fracture with small pneumocranium. Postoperatively, a prophylactic antibiotic was given for seven days and he was discharged well. Subsequent clinic visits up to one-year postoperative period showed no recurrence of the CSF leak. History taking, physical examination and CT imaging give valuable diagnostic values in managing the penetrating skull base injury. Early intervention for removal of the foreign body and repair of the CSF leak is advocated to prevent catastrophic complication.

## Introduction

Presence of fistulous tract in between intracranium and nasal cavity may result in cerebrospinal fluid (CSF) rhinorrhea. Craniofacial trauma contributed 80 % of the etiologies of CSF rhinorrhea. Traumatic CSF rhinorrhea is more commonly seen in skull base fractures. Majority of the skull base fractures (90 %) are caused by closed head injuries, whereas penetrating trauma constitutes the remaining 10 % of the etiology.

Most of the penetrating head injuries involved oro-cranial or transorbital intracranial penetration of the sharp object with a significant external injury. Foreign bodies such as glass, air gun projectiles, metal splinters and plastic chair glides have been reported. Traumatic transnasal penetrating injury to the skull base is rare in literature. Removal of the penetrating object followed by repair of the CSF leakage depicts more challenging management.

In this case report, we described an unusual case of penetrating injury of the anterior skull base by a pencil via the left nostril, without external, orbital and paranasal sinuses involvement. The importance of radiological assessment prior to surgery was emphasized in this report. We also discussed the approaches of removal of the penetrating object and endoscopic CSF leakage repair in the same setting.

## Case Report

**Figure 1:**
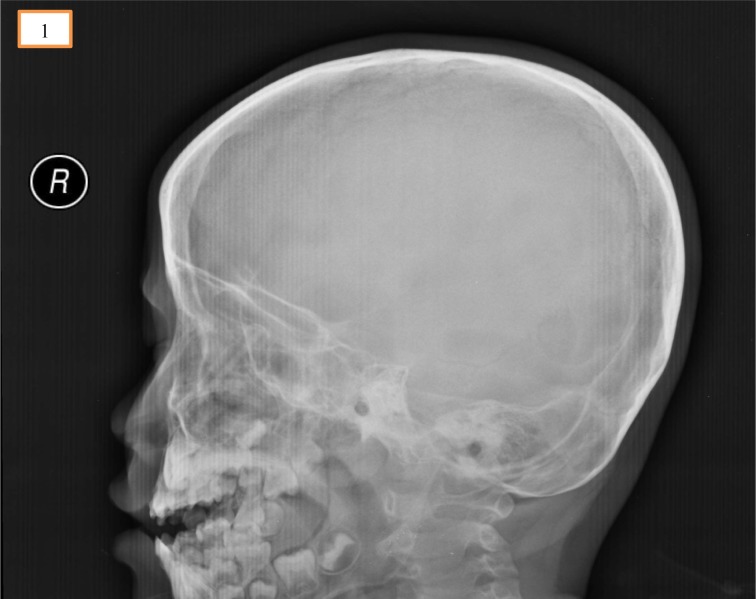
Radiograph of the skull showed faint pencil shadow over the left nasal cavity projecting towards the anterior skull base.

A 5-year-old boy presented to the emergency room with epistaxis following an impacted pencil over the left nostril. He admitted to inserting the blunted end into his left nostril and was pushed by his sibling from behind. He fell forward on his face causing impaction of the pencil into his left nostril. His mother claimed that they have successfully pulled out the pencil and the epistaxis subsequently resolved. Upon review, he was hemodynamically stable, alert and conscious. Anterior rhinoscopy showed part of the pencil shaft embedded in the left nasal cavity. The child was however uncooperative for further assessment of the extent of the injury. Otherwise, there was no external wound and deformity over the craniofacial region, bilateral eye examination and neurological examinations were unremarkable. Radiograph of the skull (Figure 1(Fig. 1)) showed faint pencil shadow over the left nasal cavity projecting towards the anterior skull base.

**Figure 2:**
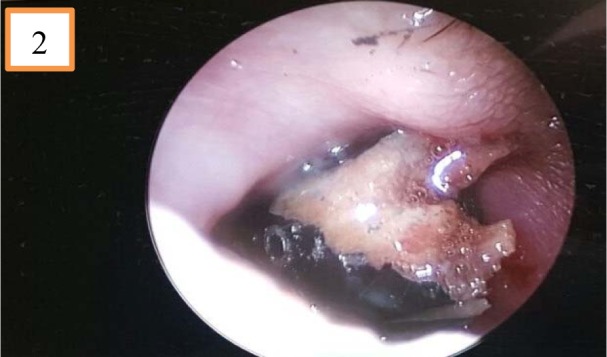
Endoscopic view of the left nasal cavity showed the pencil was impacted in the left nostril and nasal septum.

**Figure 3:**
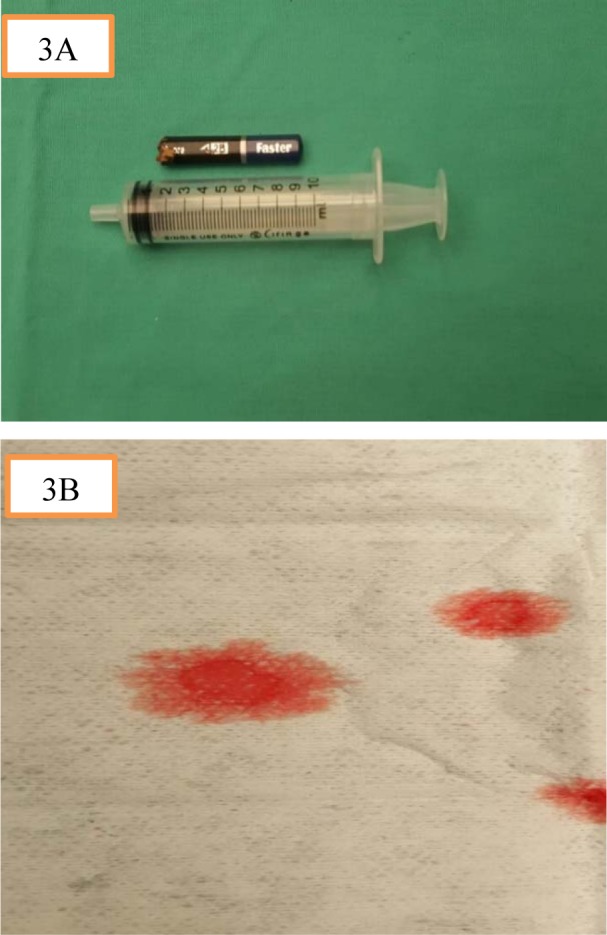
(A) The embedded pencil measured 8 cm in length and 1 cm in diameter. (B) Pulsatile clear fluid from the skull base with positive 'Halo' sign on filter paper further confirmed the skull base fracture with CSF leak.

He was brought into the operating theatre for examination under anesthesia. Intraoperatively, the pencil was impacted firmly in the left nostril and nasal septum (Figure 2(Fig. 2)). The pencil was removed with ease using a Tilley's forceps. There was a posterior nasal septal perforation following the removal of the pencil. The embedded pencil measured 8 cm in length and 1 cm in diameter (Figure 3A(Fig. 3)). An indentation mark with part of the lacerated septal cartilage was found covering the anterior skull base. Pulsatile clear fluid from the skull base was demonstrated immediately after removal of the pencil further which confirmed the skull base fracture with CSF leak (Figure 3B(Fig. 3)). We postulated that the blunt end of the pencil served as the entry point via left nostril, penetrated the nasal septum with the extension of the injury to the anterior skull base.

After removal of the foreign body, the CSF leakage was repaired with autologous graft by placing the septal cartilage and abdominal fat on to the defect until the CSF leakage ceased. The septal mucosal graft was repositioned on top of the fat graft and covered with Gelfoam and tissue glue.

**Figure 4:**
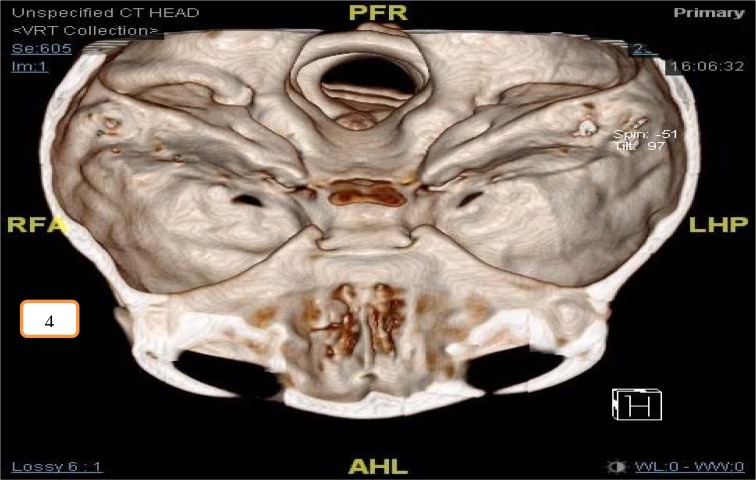
Three-dimensional (3D) images reconstructed from the fine cut CT scan of the brain, skull base and paranasal sinuses showed right cribriform plate fracture.

Post-operatively, he was ventilated for 24 hours and kept under bed rest with intravenous ceftriaxone administered for one week in view of overwhelming intraoperative findings. Computed Tomography (CT) scan of the brain, skull base and paranasal sinuses post-operatively showed right cribriform plate fracture with small pneumocranium (Figure 4(Fig. 4)). No evidence of intracranial hemorrhage was seen. Post-extubation, his pupils were equal and reactive bilaterally with no neurological deficits. The neurosurgical team suggested for conservative management. He was discharged well one-week post-surgery. Subsequent nasendoscopy during regular outpatient visits up to one year did not show any CSF leak.

## Discussion

Nasal foreign bodies are common encounter in ENT setting. It ranges from simple foreign bodies to catastrophic scenarios with skull base fractures and intracranial complications.

Transnasal septal penetrating injury to the anterior skull base without an external wound and orbital involvement makes this a unique case. Furthermore, management of penetrating injury of the head and neck region in a pediatric patient can be very challenging as history may be inaccurate with limited cooperation during an examination. Additionally, clinical examination showed no external wound over the face and orbital region with intact neurological function masquerade the extent of the injury made.

When the history is uncertain, the evaluation of suspected penetrating injury of the nose lies on the radiological assessment. A plain radiograph is easily available with low radiation, it offers an initial assessment of the bony framework of the facial and skull bone to detect any fracture lines. The panoramic view of plain radiograph helps in locating the foreign body. However, the visibility of the foreign body in plain radiograph depends on the types of object. Metallic foreign body is radio-opaque on radiograph whereas organic foreign body like peanut or wood would be less radio-opaque hence easily missed (Zilinskiene et al., 2014[10])*.*

CT scan gives a good osseous delineation which is important in detecting subtle fractures. This fine thin cut of the CT scan with the image in axial, coronal and sagittal plane provides an accurate assessment of the location of the foreign body. With the three-dimensional (3D) images reconstructed from the fine cut CT scan, the extent of the injury can be accurately localized and essential in planning the surgical approach. For cases suspected with vasculature or intracranial injury, a CT angiography and Magnetic Resonance Imaging (MRI) brain would be warranted (Tsao et al, 2006[8]).

Early removal of the penetrating foreign body is the definitive treatment in regards to avoid the unwanted sequelae (Brinson et al., 2004[2]). The foreign body in the nasal cavity will obstruct the drainage and ventilation of the paranasal sinuses eventually causing sinusitis. Penetration of the foreign body into the skull base with CSF leak predisposes the patient with intracranial infection by direct bacterial inoculation or ascending infection via the defects on the skull base (Cho et al., 2016[3]). Early removal of foreign body and repair will reduce the risk of meningitis and cerebral abscess (Hettige et al., 2010[4]). The patient was hemodynamically stable and surgical removal was carried out without further delay. He was given seven days of intravenous ceftriaxone, which is the 3^rd^ generation of cephalosporin with the property of crossing blood-brain barrier for prophylaxis against intracranial infection.

There are two suggested methods in the removal of the penetrating foreign body into the maxillofacial region, namely the endonasal approach and external approach (Tsao et al., 2006[8]). The shape and size of the object and the location of the entry point must be taken into consideration when planning for the removal. The penetrating object in this case report was a long and straight pencil with the entry point at the left nostril. This is best to be removed with endonasal approach via the natural orifice without leaving any additional scar on the face.

CSF leak was demonstrated immediately after removal of the foreign body. Most of the traumatic CSF leak will be resolved with conservative management or CSF diversion with lumbar drain. However, the incidence of ascending intracranial infection was relatively high in the group of CSF leak patient when conservative treatment was given. Thus, repair of the CSF leak was advocated whereby it significantly reduces the risk of ascending intracranial infection (Bernal-Sprekelsen et al., 2005[1]). 

Presence of fistulous tract in between intracranial and nasal cavity predisposes the patient to CSF leaks, pneumocephalus and intracranial infection such as ascending meningitis and abscess. The goals of skull base reconstruction include separation of the cranial cavity from the sinonasal tract, protection of neurovascular structures, preservation of functions and avoidance of dead spaces.

As stated by Wormald and McDonough in 1997[9], CSF leaks have been traditionally managed by neurosurgeons via a frontal craniotomy. This traditional approach has a success rate of 60-80 % but has also been associated with high morbidities such as frontal lobe retraction and anosmia. With the advancement of endoscopy technique, Oskar Hirsch from Austria was the first to use a strictly endonasal approach to close a CSF leak in sphenoid sinus using a mucosal perichondrial flap from nasal septum via a transseptal-transsphenoidal approach in 1952 (Hirsch, 1952[5]).

Indications for endoscopic repair include post-traumatic CSF leak, iatrogenic and spontaneous CSF rhinorrhea associated with either malformation of the skull base, meningoencephaloceles, empty sella and increased intracranial pressure. With the improvement of skills and instrumentation, amazingly high success rates with primary closure of the defect between 88 % and 94 % were achieved in the early 1990s (Lund et al., 2010[7]).

The key to successful management of CSF leak is to precisely identify the site of the dural tear. Preoperative imaging such as high-resolution CT scan of the skull base and paranasal sinuses can be performed to locate the fracture site. Intrathecal application of sodium fluorescein in combination with the use of blue light and a blocking filter over the endoscope's eyepiece will precisely guide the endoscopic surgeon to the site of the lesions. Laboratory techniques such as Beta-2-transferrin and beta-trace protein testing are the standard tests that help to differentiate non-CSF-related nasal secretion from the true CSF in an unclear situation (Le et al., 2016[6]).

Multiple reports have validated that small CSF fistula can be reconstructed with a variety of free grafting techniques achieving success rate in more than 95 % of patients. In this case, the skull base defect was repaired via a transnasal endoscopic method by using autologous grafting. Examples of autologous grafts are fascia lata, fascia temporalis, cartilage, bone and fat. The autologous graft is preferred as these avoid all the potential risks of heterogenous grafts like a prion-associated disease, HIV, hepatitis and other transmittable disease entities (Lund et al., 2010[7]).

In this case, we harvested the fat graft from the periumbilical region as the autologous graft for repair. The indentation mark over the anterior skull base was covered by the lacerated septal cartilage with septal mucosa. For defect closure, it is recommended that no mucosa should be placed under any graft or fat to avoid mucocele formation. Hence the septal cartilage with mucosa was taken out temporary, the area of skull base defect was denuded of its mucosal layer. Adequate size of the harvested fat graft was gently placed over the skull base defect with pediatric Blakesley forcep. With the use of Freers elevator, the fat graft was gently placed in between the bone and surrounding mucosa, which is described as overlay technique. Subsequently, the septal cartilage with mucosa was repositioned back on top of the fat graft. The grafted area was reinforced with Gelfoam and tissue glue. The end result of the repair was excellent without intracranial complication and further CSF leak up to date.

## Conclusion

Management of transnasal penetrating injury of the anterior skull base can be very challenging in terms of diagnosis and treatment. When the history and physical examination are in doubts, CT imaging is mandatory to rule out suspecting skull base injury. Having said that accurate diagnosis and early intervention are important in order to prevent intracranial complications, continuous follow up is advocated to detect the delayed complication.

## Conflict of interest

The authors declare no conflict of interest.
